# Suicide and Ageism

**DOI:** 10.1192/j.eurpsy.2022.114

**Published:** 2022-09-01

**Authors:** D. De Leo

**Affiliations:** Griffith University, Australian Institute For Suicide Research And Prevention, Brisbane, Australia

**Keywords:** Suicide, prevention, ageism, old age

## Abstract

Suicide in old age is frequently considered the result of a rational decision. Accumulation of physical illnesses, frailty, dependence on others, loss of partner and loneliness are often seen as reasons that might justify suicidal acts. Depression is an important risk factor for suicidal behaviour even at very advanced age. However, ageistic views tend to consider depression as a normal feature of the aging process; it is possible that its presence can be overestimated or perhaps generalized more than necessary by making it the scapegoat of any situation related to suicide. In fact, adopting an attitude that involves excessive simplification of problems, where everything is attributable to ‘depression’, can induce a rigid prescriptive approach, often limited to the indication of an antidepressant drug. In this way, the appreciation of the multifactorial nature of an individual’s crisis becomes too narrow and the chances of counteracting the complexities of a dangerous suicide progression too modest. From a prospect of suicide prevention, approaching a patient carefully and prudently is always to be preferred to the disposition that considers life events as inevitable as well as all reactions related to them, including the most extreme, such as suicide. This attitude can lead to a poor involvement of the treating physicians, who might become too acceptant of the ‘unavoidability’ of the unfavourable progression of their patient. Such a passive approach may especially characterise the relationship with patients of very advanced age, where stressors of physical and non-physical nature easily aggregate, multiplying their impacting power.

**Disclosure:**

No significant relationships.

